# Interpretable machine learning for the prediction of death risk in patients with acute diquat poisoning

**DOI:** 10.1038/s41598-024-67257-6

**Published:** 2024-07-12

**Authors:** Huiyi Li, Zheng Liu, Wenming Sun, Tiegang Li, Xuesong Dong

**Affiliations:** 1 Department of Emergency Medicine, The First Hospital of China Medical University, Shenyang, China; 2 Department of Emergency Medicine, Shengjing Hospital of China Medical University, Shenyang, China

**Keywords:** Diquat poisoning, Risk of death, Machine learning, Shapley additive explanations, Medical research, Risk factors

## Abstract

The aim of this study was to develop and validate predictive models for assessing the risk of death in patients with acute diquat (DQ) poisoning using innovative machine learning techniques. Additionally, predictive models were evaluated through the application of SHapley Additive ExPlanations (SHAP). A total of 201 consecutive patients from the emergency departments of the First Hospital and Shengjing Hospital of China Medical University admitted for deliberate oral intake of DQ from February 2018 to August 2023 were analysed. The initial clinical data of the patients with acute DQ poisoning were collected. Machine learning methods such as logistic regression, random forest, support vector machine (SVM), and gradient boosting were applied to build the prediction models. The whole sample was split into a training set and a test set at a ratio of 8:2. The performances of these models were assessed in terms of discrimination, calibration, and clinical decision curve analysis (DCA). We also used the SHAP interpretation tool to provide an intuitive explanation of the risk of death in patients with DQ poisoning. Logistic regression, random forest, SVM, and gradient boosting models were established, and the areas under the receiver operating characteristic curves (AUCs) were 0.91, 0.98, 0.96 and 0.94, respectively. The net benefits were similar across all four models. The four machine learning models can be reliable tools for predicting death risk in patients with acute DQ poisoning. Their combination with SHAP provides explanations for individualized risk prediction, increasing the model transparency.

## Introduction

Over the past few decades, there has been a global increase in the use of pesticides, especially herbicides. The mortality rate of patients with acute oral paraquat (PQ) poisoning due to the absence of specific detoxification drugs is as high as 60–70%, making PQ the most deadly pesticide poisoning event at present^1^. PQ is banned in many countries. As a substitute^2^, diquat (1,1′-ethylene-2,2′-bipyridinium, DQ) is a widely used herbicide in agriculture, which has caused an increase in the number of clinical patients with DQ poisoning annually^3^. DQ is a nonselective, defoliant, preharvest, desiccant herbicide of the bipyridinium class. It was first synthesized by Dr. Fielden at the Dyestuffs Division laboratories of the Imperial Chemical Industries (Blackley, England). DQ was first introduced to the market by Imperial Chemical Industries in 1958. Due to the lack of specific antidotes for DQ poisoning, the clinical treatment of DQ poisoning has not been ideal; its mortality rate is high, and this is one of the current challenges in the treatment of DQ poisoning^4^. To remove DQ from the body, several methods, including forced diuresis, haemoperfusion, and extracorporeal haemodialysis, have been explored. However, the effectiveness of these techniques has been questioned in certain studies, as they may not eliminate substantial amounts of clinically and toxicologically significant herbicides. This limitation is likely attributed to the rapid dispersal of absorbed DQ^5^. Currently, there are only a few publications on DQ than PQ, especially clinical prognostic studies^6–8^. The studies on DQ have mainly comprised case reports and mechanistic studies^4,9–11^, and there are no reported investigations of clinical outcomes. Therefore, if the risk factors for death can be identified and the early admission prognosis can be accurately predicted, the treatment of patients can be improved and optimized.

Recently, machine learning has attracted the attention of clinicians and has gained their recognition because of the evolution of statistical theory and computer technology. Novel machine learning techniques have been widely used in predictive models of various diseases and have shown better performance than traditional logistic regression analyses^12,13^. Machine learning approaches offer advantages in that they account for high-order, nonlinear interactions between predictors and achieve more stable predictions. In recent years, statistical prediction models across the majority of disease types have been developed. The advent of machine learning models has shown promise for improving the predictive ability of various conditions (e.g., sepsis and emergency department triage)^14,15^. Machine learning has generally not been applied to poisoning-related diseases. Due to the “black box” nature of machine learning, it is difficult to explain why certain predictions should be made for patients. The lack of interpretability limits its use in medical decision support. SHapley Additive exPlanations (SHAP) can precisely calculate the contribution and impact of each feature on the final prediction, serving as a new method for interpreting various machine learning models. We combined four common machine learning methods with SHAP based on early admission indicators of patients with DQ poisoning to construct an interpretable predictive model that is aimed to assist clinicians in the early prognosis assessment of patients.

## Results

### Baseline characteristics

Among 201 patients (106 [52.71%] male), the median (IQR) age was 33 (23–48), and 100 patients were dead. The differences in patient characteristics between the survival and nonsurvival groups are described in Table 1. The results showed that compared with those in the nonsurviving group, the age, WBC, ALT, TBil, DBil, BUN, Cr, Glu, TnI, BNP, PaCO_2_, lactic acid and DQd in the surviving group were significantly different (all p < 0.05). There was no significant difference in sex, Hb, PLT, K^+^, ALB, pH, PaO_2_, haemoperfusion or shock index (all p > 0.05).

**Table 1:** Baseline characteristics of the patients with survival and nonsurvival.

Item	Overall (n = 201)	Survival group (n = 101)	Nonsurvival group (n = 100)	*P*
Male [n (%)]	106(52.71)	49 (48.51)	57 (57)	0.23
Age (year)	33(23–48)	30.00 (22.00–40.00)	38.00 (25.00–54.00)	0.001
WBC (×10^9^/L)	16.25 ± 8.18	12.08 ± 5.12	20.46 ± 8.54	< 0.001
Hb (g/L)	146.87 ± 17.89	144 ± 14.92	148.79 ± 20.36	0.119
PLT (×10^9^/L)	264.94 ± 81.29	257.78 ± 66.75	272.18 ± 93.52	0.436
ALT (U/L)	24 (15–40)	19 (14–30)	30 (18.25–72.5)	< 0.001
TBil (μmol/L)	12.4 (9.1–18)	11.70 (8.80–14.95)	12.80 (9.28–21.20)	0.035
DBil (μmol/L)	4.4 (2.9–6.0)	4.00 (2.44–5.45)	4.55 (3.00–7.50)	0.021
ALB (g/L)	46.56 ± 5.54	46.56 ± 4.55	46.56 ± 6.41	0.457
K^+^ (mmol/L)	3.842 ± 0.541	3.88 ± 0.38	3.81 ± 0.67	0.070
BUN (mmol/L)	5.20 (3.89–6.70)	4.62 (3.63–5.72)	5.85 (4.3–7.80)	< 0.001
Cr (µmol/L)	70.5 (56–112)	58 (47.4–69.55)	103.2 (70.63–168.48)	< 0.001
Glu (mmol/L)	6.84 (5.80–8.30)	6.17 (5.41–7.42)	7.5 (6.33–8.98)	< 0.001
TnI (ng/mL)	0.01 (0.00–0.01)	0.00 (0.00–0.01)	0.01 (0.00–0.04)	< 0.001
BNP (pg/mL)	10 (10–53)	10.00 (10.00–33.75)	14.15 (10.00–99.33)	0.009
PH	7.40 (7.36–7.44)	7.40 (7.38–7.44)	7.40 (7.35–7.44)	0.135
PaO_2_ (mmHg)	106 (90–128.5)	103.00 (90.70–125.50)	109.95 (82.41–130.63)	0.956
PaCO_2_ (mmHg)	29.85 ± 7.35	34.03 ± 4.77	25.62 ± 7.10	< 0.001
Lactic acid (mmol/L)	2.02 (1.30–4.90)	1.5 (1.1–1.85)	4.35 (2.33–6.7)	< 0.001
Haemoperfusion [n (%)]	155 (77.11)	79 (78.22)	76 (76)	0.709
DQd (mL)	100 (30–200)	30 (15–65)	160 (100–200)	< 0.001
Shock index	0.68 (0.58–0.81)	0.7 (0.6–0.8)	0.7 (0.53–0.9)	0.354

WBC, white blood cell; Hb, haemoglobin; PLT, platelet; ALT, alanine aminotransferase; TBil, total bilirubin; DBil,

direct bilirubin; ALB, albumin; K^+^, potassium; BUN, blood urea nitrogen; Cr, creatinine; Glu, glucose; TnI, troponin I;

BNP, brain natriuretic peptide; PaO_2_, partial pressure of oxygen; PaCO_2_, partial pressure of carbon dioxide; DQd, diquat dose.

### Model performance comparisons

**Figure 1:**
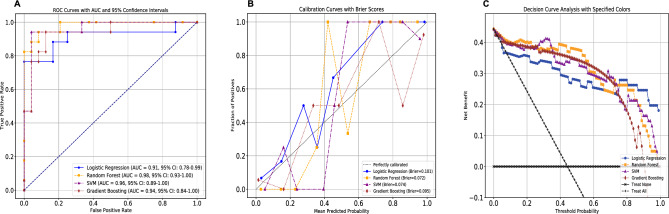
The performance of the models. SVM, support vector machine. (**A**) The receiver operating characteristic curves of the four models. (**B**) The calibration curves of the models. (**C**) The decision curve analyses of the four models.

We generated four machine learning models to predict the risk of death in patients with acute DQ poisoning. The results show the discriminative performance of the four models in terms of ROC curves. Among the four models, the random forest model (AUC = 0.98) had the best predictive effect for death risk in patients with acute DQ poisoning, followed by the SVM model (AUC = 0.96), gradient boosting model (AUC = 0.94) and logistic regression (AUC = 0.91), as shown in Fig. 1A. The performance of the four models is shown in Table 2, with the random forest model achieving the highest F1-score (0.90), the highest MCC (0.79), the highest accuracy (0.90), and the lowest Brier score (0.07). The calibration curve is shown in Fig. 1B. With the exception of gradient boosting, whose Hosmer‒Lemeshow χ2 value was 27.84 (p < 0.001), indicating poor calibration, the remaining three models all demonstrated good calibration, as shown in Table 2. (A significant test statistic implies that the model does not calibrate perfectly^16^). According to the DCA curve, all models provided decent net benefits, as shown in Fig. 1C, with similar net benefits at the 5% decision threshold, as shown in Table 2.

**Table 2:** Performance of the four models.

Models	Overall					Discrimination	Calibration		Clinical usefulness
	F1-score	MCC	Accuracy	Precision	Recall	AUC (95%CI)	Hosmer–Lemeshow χ^2^ (*p*)	Brier score	Net benefit at threshold of 5%
Logistic regression	0.82	0.71	0.85	1.00	0.65	0.91 (0.78–0.99)	11.92 (0.155)	0.10	0.41
Random forest	0.90	0.79	0.90	0.88	0.88	0.98 (0.93–1.00)	10.02 (0.264)	0.07	0.42
SVM	0.87	0.75	0.87	0.93	0.76	0.87 (0.89–1.00)	2.56 (0.959)	0.09	0.43
Gradient boosting	0.87	0.74	0.87	0.89	0.82	0.94 (0.84–1.00)	27.84 (< 0.001)	0.09	0.40

AUC*,* area under the curve; MCC*,* Matthews correlation coefficient; SVM, support vector machine.

### Feature importance and model interpretation

**Figure 2:**
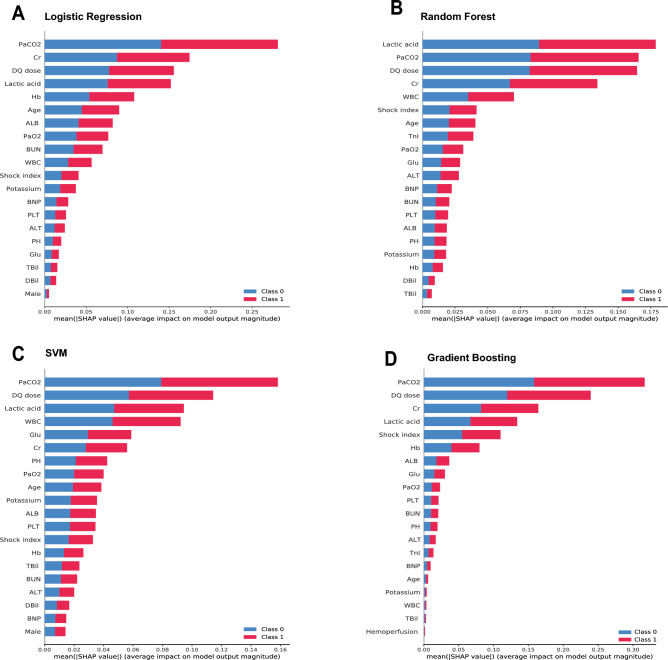
SHapley Additive exPlanation (SHAP) values of the main features of the models**.** SVM, support vector machine; WBC, white blood cell; Hb, haemoglobin; PLT, platelet; ALT, alanine aminotransferase; TBil, total bilirubin; DBil, direct bilirubin; ALB, albumin; K^+^, potassium; BUN, blood urea nitrogen; Cr, creatinine; Glu, glucose; TnI, troponin I; BNP, brain natriuretic peptide; PaO_2_, partial pressure of oxygen; PaCO_2_, partial pressure of carbon dioxide; DQ dose, diquat dose. Class 0 represents surviving patients, and Class 1 represents nonsurviving patients.

**Figure 3:**
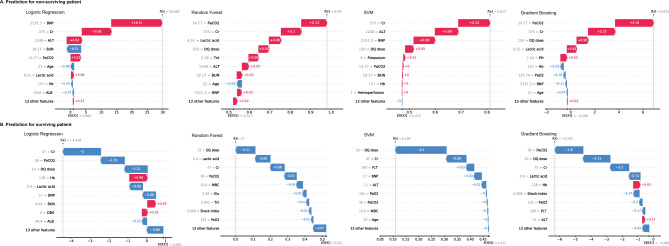
Interpretation of the predicted results for the two selected patients by all four models. SVM, support vector machine; WBC, white blood cell; Hb, haemoglobin; PLT, platelet; ALT, alanine aminotransferase; ALB, albumin; BUN, blood urea nitrogen; Cr, creatinine; Glu, glucose; TnI, troponin I; BNP, brain natriuretic peptide; PaCO_2_, partial pressure of carbon dioxide; DQ dose, diquat dose.

The SHAP were calculated to assess the importance of each feature. This process requires sequentially integrating features, starting with the most important feature, and gradually adding the next feature in order of importance^17^. The contributions of all the features were essentially equal across the four models for survival (class = 0) and nonsurvival (class = 1) (Fig. 2). Figure 3 shows the bar graphs of the predictions for nonsurviving and surviving patients. F(x) is the log odds ratio for each observation. The arrows indicate the impact of each factor on the prediction. The blue and red arrows represent whether the factor decreased (blue) or increased (red) the risk of death, respectively. The longer the arrow is, the greater the effect.

## Discussion

This study effectively predicted the risk of death in patients with acute DQ poisoning using interpretable machine learning methods and common clinical indicators. As the use of PQ has gradually decreased, the incidence of poisoning by its substitute herbicide, DQ, has gradually increased. Currently, there is no specific antidote for DQ poisoning; therefore, the death rate of poisoned patients is high^4^. Clinicians face great challenges in both the assessment and clinical treatment of such poisoning. Therefore, obtaining a simple and intuitive assessment method is highly important for quickly identifying the risk of death in acute and critical patients with rapid DQ intoxication.

DQ is a potent redox cycler that is readily converted to a free radical, which, when reacted with molecular oxygen, generates superoxide anions and, subsequently, other redox products. These products can induce lipid peroxidation in cell membranes and potentially lead to cell death^18^. When DQ enters the body, it is reduced by receiving a single electron from NADPH, which is the primary source of reducing equivalents in cells, forming NADP^+^ and a highly unstable DQ^+·^. In turn, DQ^+·^ transfers an electron to molecular oxygen (O_2_) to generate O_2_^·+^.

DQ^+·^ can revert to its initial state and undergo this continuous process to generate large quantities of O_2_^+·^. This O_2_^+·^ is subsequently neutralized spontaneously or through superoxide dismutase (SOD) activity, resulting in the formation of hydrogen peroxide (H_2_O_2_) and O_2_^19^. Under normal circumstances, H_2_O_2_ is converted to water through the action of catalase and glutathione peroxidase. However, in the presence of a substantial increase in reactive oxygen species production, the defence mechanisms within the cell, such as nonenzymatic constituents or antioxidant enzymes, are overburdened, leading to oxidative stress. Consequently, cellular dysfunction and injury occur^20–22^.

DQ is believed to significantly affect hepatic and renal toxicity through the involvement of free radicals^21^. This compound specifically induces damage to the kidney by affecting its excretory function, leading to conditions such as oliguria, anuria, proteinuria, haematuria, pyuria, azotemia, acute renal failure, and acute tubular necrosis^23,24^. In this study, consistent with previous findings, renal impairment was found to be a risk factor for death in patients with acute DQ poisoning. At the same time, DQ can also damage the liver, central nervous system, lungs, etc., as well as damage to the local reproductive system and the skin have also been reported^3,4,25^. Dyspnoea, pulmonary oedema, and respiratory depression are manifestations of pulmonary injury. However, unlike for PQ poisoning, there are no reports of pulmonary fibrosis caused by DQ poisoning^26,27^. In fact, in animal experiments, DQ caused mild and reversible damage to type I alveolar epithelial cells but not to type II alveolar epithelial cells^28^. Currently, there are no known remedies or successful treatments for DQ poisoning, and the focus of treatment has been on minimizing absorption and/or improving elimination^18,29^.

This study is the first to apply machine learning to predict the risk of death from acute DQ poisoning. Machine learning models are widely used in clinical diagnostics, precision treatments, and health monitoring and have achieved good results^30,31^. Each model has its own advantages and disadvantages. For example, Random Forest has the benefit of fewer predictor variable assumptions than traditional modelling strategies and has minimal overfitting compared to simple classification and regression trees. However, Random Forest model has the fundamental issue of being a black box model. When alarms sound, medical staff are unsure of what immediate action to take until the patient is checked (cannot describe relationships within data^32^). In this study, we employed machine learning combined with SHAP to assess the risk of death in patients with acute DQ poisoning. Previous studies primarily relied on logistic regression analysis and have not yet explored the application of machine learning. Consequently, there remains a dearth of evidence regarding the benefits of machine learning in predicting the risk of death in patients with DQ poisoning. Our results demonstrate that all four models exhibit strong performance, with Random Forest surpassing traditional logistic regression analysis in terms of efficiency, as indicated by the ROC curves. We further plotted the importance features of random forest. The results revealed that Cr, PaCO_2_, DQd, lactic acid, and WBC were important features for predicting death in patients with acute DQ poisoning. Higher levels of Cr, lactic acid, oral dosage of DQ, and WBC were associated with an increased risk of death, while lower levels of PaCO_2_ were also correlated with a greater risk of death. Most poisoning cases are related to the intentional ingestion of concentrated liquid formulations. In this study, the results showed a direct relationship between DQ intake and patient death. With the increase in the oral dose of DQ, the death rate of patients increased significantly, consistent with the results of previous studies^33^ that have shown that the ingestion of more than 15 mL of a rapid dose of 20% concentrated formulation of DQ is usually fatal.

The results of this study showed that the higher the lactic acid concentration, the greater was the risk of death. The prognostic ability of arterial lactate levels has been assessed in various critical care patient groups, including those with septic shock, circulatory shock, recent surgical procedures, burns, and trauma. The level of lactic acid has emerged as a reliable predictor of mortality in individuals with severe illness^34^. In previous studies on the prognosis of acute PQ poisoning, clinical cases from different countries have shown that lactic acid is a good predictive factor^35,36^.

In this study, a lower PaCO_2_ suggested a greater risk of death. In one study, a decrease in PaCO_2_ caused cerebral vasoconstriction, with a 1 mmHg change in PaCO_2_ corresponding to a decrease in cerebral blood flow of 1.8 mL/100 g/min^37^. According to results, the WBC count is associated with poor outcomes. Many toxic diseases, such as acute organophosphate insecticide poisoning (AOPP), increase the WBC count, making it a poor indicator of prognosis^38^. In previous studies, in patients with acute PQ poisoning, an elevated WBC count was one of the indicators of poor prognosis^39^.

The random forest model had a higher F1-score, accuracy, AUC, and MCC, and the Brier score was also the lowest. Compared to the other models, its overall performance was slightly better. DCA demonstrated that the four models provided a good net benefit within a range of thresholds (Fig. 1C). Overall, all the four models demonstrated good predictive performance, with Random Forest performing slightly better.

The SHAP calculation method was used in this study, which shows a list of important features, from most important to least important (from top to bottom). All the features contributed equally to the prediction of nonsurvival and survival, but the feature weights contained in the different models were not the same (Fig. 2). We provided two examples to illustrate the interpretability of the model, one for a nonsurviving patient and one for a surviving patient (Fig. 3). All four models presented very consistent predictive results in a straightforward manner, enabling clinicians to clearly observe the weights contributed by the included features in the model predictions. Individual predictors are greatly influenced by subjective factors; for example, the oral dose of patients is subjective and may not be very accurate, and vomiting dose, gastric lavage time, etc., affect the actual amount of absorption. Most earlier studies included only the patient’s clinical test indicators and not their vital signs. This study combined objective indicators and patient status to objectively and intuitively evaluate the prognosis of patients with acute DQ poisoning.

### Limitations

The sample size was small, which may have led to bias. In the future, we hope to continue to expand the sample size, summarize previous research experience, and strengthen the cooperation between basic and clinical studies to carry out high-quality clinical research for further demonstration. This research was based on a retrospective analysis; here, data were acquired from two distinct medical facilities, but due to limited data availability, the samples could not be divided into a testing group. Consequently, external validation is necessary to further evaluate the performance of our results.

## Conclusion

Our study indicates that machine learning can accurately assess the risk of death in patients with acute DQ poisoning. Combining machine learning with SHAP provides clear explanations for individualized risk prediction, enabling physicians to intuitively understand the impact of key features in the model.

## Methods

### Source of data

This was a retrospective multicentre study, and the study design followed the Transparent Reporting of a Multivariable Prediction Model for Individual Prognosis or Diagnosis (TRIPOD) reporting guidelines. From February 2018 to August 2023, 201 consecutive patients with deliberate oral DQ poisoning were retrospectively reviewed; these included 93 patients from the emergency department of the First Hospital and 108 patients from the emergency department of Shengjing Hospital of China Medical University. The study protocol was approved by the Ethics Committee of the First Hospital of China Medical University (approval no. 2023[330]). All the data were analysed anonymously, and the need to obtain informed consent from the patients was waived.

### Study population and definition of outcome

The inclusion criteria were as follows: patients admitted for deliberate oral intake of DQ within 24 h, patients aged > 14 years, and patients whose haemoperfusion was not performed before presentation. Patients who had severe chronic comorbidities, including symptomatic heart failure, decompensated liver cirrhosis, chronic obstructive pulmonary disease, or chronic kidney disease, or who received dialysis treatment before admission were excluded. In-hospital death was considered the endpoint, and the patients were categorized into a survival group and a nonsurvival group. In addition to an evaluation of each patient’s main complaint, the diagnosis of DQ was confirmed by urine colorimetric analysis, and patients with PQ intoxication or mixed intoxication with PQ were excluded. In emergency situations, when DQ poisoning is suspected, a rapid and simple colorimetric test can be performed by analysing urine by adding sodium bicarbonate or hydroxide, followed by sodium dithionite powder, which results in a green colour in the presence of DQ^4^.

### Feature selection and data preprocessing

**Figure 4:**
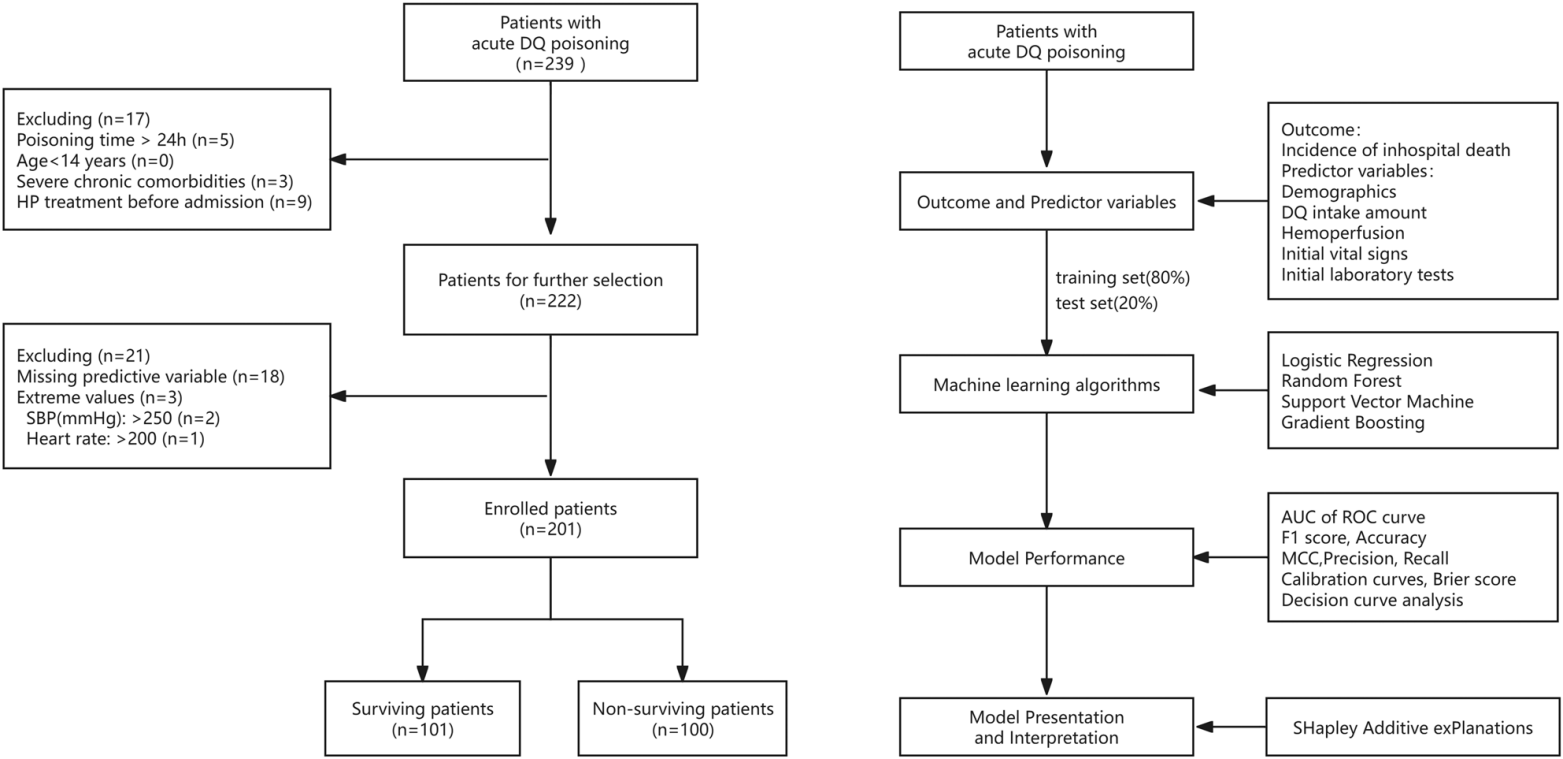
The flowchart of the study**.** DQ, diquat; HP, haemoperfusion; SBP, systolic blood pressure; MCC, matthews correlation coefficient.

The following data of all patients were recorded in the medical record system: (a) demographic parameters, such as age and sex; (b) the estimated DQ intake dose, whether haemoperfusion was performed; (c) vital data, including the shock index (pulse/systolic blood pressure) and oxygen saturation, which were were recorded upon first admission; and (d) blood biochemical indicators, including white blood cell (WBC) count, haemoglobin (Hb), platelet (PLT), alanine aminotransferase (ALT), total bilirubin (TBil), direct bilirubin (DBil), albumin (ALB), potassium (K^+^), blood urea nitrogen (BUN), creatinine (Cr), glucose (Glu), troponin I (TnI), brain natriuretic peptide (BNP), pH, partial pressure of oxygen (PaO_2_), partial pressure of carbon dioxide (PaCO_2_), and lactic acid, which were measured at the first admission. To improve the accuracy of the model, we used a normalization method to scale all the variables and map the data to the [0,1] interval. Missing and extreme values were deleted, and no imputation was performed. In this study, there were few missing values and outliers. Considering the modelling accuracy, missing values and outliers were deleted rather than imputed, as shown in Fig. 4.

### Statistical analysis

Continuous variables are presented herein as the means (SDs) or medians (IQRs). For comparisons according to their suitability, Student’s t test or the Mann‒Whitney U test was used. Categorical variables are presented as numbers (percentages) and were compared with the χ^2^ test.

Four machine learning methods, namely, logistic regression, random forest, support vector machine (SVM) and gradient boosting, were employed for model construction. The samples were randomized into a training set (80%) and a test set (20%). Subsequently, the performance of each model was validated and compared using the test set. In our study, the model with the highest area under the curve (AUC) of the receiver operating characteristic (ROC) curve was selected as the optimal model. The 95% confidence interval (CI) for the area under the curve (AUC) was calculated using the bootstrap method (1000 iterations). Next, calibration curves were plotted to assess the calibration of the four models, accompanied by the Hosmer–Lemeshow test. We calculated the F1 score, accuracy, Matthews correlation coefficient (MCC), precision, recall and Brier score. The net benefit of patients was evaluated through clinical decision curve analysis (DCA). SHAP were used to explain model features and, combined with examples, demonstrated the contribution (positive or negative) of predictive variables to the target variable. The study process is shown in Fig. 4.

The statistical analyses and graphics were performed using IBM SPSS (22.0) and Python (3.8.5). For all the analyses, p < 0.05 was considered to indicate statistical significance, and all tests were two-tailed unless otherwise indicated. All methods described in this study were performed in accordance with the relevant guidelines^40^ and regulations. The specific packages, parameters, and code used in this study can be viewed and downloaded from GitHub (Supplementary Information).

### Supplementary Information

Supplementary Information 1.

Supplementary Information 2.

## Data Availability

Readers can publicly access the data and code for this study on our GitHub repository: https://github.com/liuzheng01/dq_data.
